# PD-1 inhibitors increase the incidence and risk of pneumonitis in cancer patients in a dose-independent manner: a meta-analysis

**DOI:** 10.1038/srep44173

**Published:** 2017-03-08

**Authors:** Jiaying Wu, Dongsheng Hong, Xiangnan Zhang, Xiaoyang Lu, Jing Miao

**Affiliations:** 1 The First Affiliated Hospital, College of Medicine, Zhejiang University, Hangzhou, China; 2 Department of Pharmacology, College of Pharmaceutical Sciences, Zhejiang University, Hangzhou, China

## Abstract

Therapies that targeted PD-1 have shown remarkable rates of durable clinical responses in patients with various tumor types. However, the extent and knowledge of pulmonary toxicities associated with PD-1 blockade, mainly manifested as pneumonitis, remains obscure. In this study, a total of 6360 subjects from 16 phase II/III clinical trials were pooled for meta-analysis to evaluate the overall incidence and risk of PD-1 inhibitors-related pneumonitis in cancer patients. The incidence of pneumonitis during anti-PD-1 immunotherapy was 2.92% (95%CI: 2.18–3.90%) for all-grade and 1.53% (95%CI: 1.15–2.04%) for high-grade pneumonitis. Compared with routine chemotherapy, PD-1 inhibitors were associated with a significant increased risk of pneumonitis. Moreover, among the types of tumor treated with PD-1 inhibitors, the melanoma patients have the lowest incidence of pneumonitis, while the non-small cell lung cancer (NSCLC) and renal cell carcinoma (RCC) patients have the highest. Furthermore, no significant differences were detected in the incidences of all- and high-grade pneumonitis between high-dose and low-dose groups of PD-1 inhibitors. In conclusion, PD-1 inhibitors were probably associated with an increased risk of pneumonitis in a dose-independent manner, compared with routine chemotherapeutic agents. The frequency and severity of treatment-mediated pneumonitis was quite different in patients with various tumor types.

The programmed cell death protein 1 (PD-1, also known as PDCD1), functioning as an immune checkpoint, plays an important role in down regulating the immune system by preventing the activation of T-cells^1^,^2^,^3^,^4^. Blockade of the PD-1 pathway with antibodies could augment the function of tumor-specific CD4+ T-cells and restore the anti-tumor immunity^5^,^6^,^7^. So far the US Food and Drug Administration (FDA) has approved only two IgG4-κ monoclonal antibodies (mAbs) for PD-1 inhibition, pembrolizumab (humanised; Keytruda^®^, Merck) and nivolumab (fully human; Opdivo^®^, Bristol-Myers Squibb), for the treatment of patients with unresectable or metastatic melanoma and metastatic squamous non-small-cell lung cancer (NSCLC)^8^. Combination therapy of PD-1 inhibitor with ipilimumab, an antibody against CTLA-4, which is another immune checkpoint inhibitor, has also been found to be effective for melanoma^9^,^10^,^11^. Although anti-PD-1 immune checkpoint mAbs have demonstrated antineoplastic activity across multiple malignancies, the toxicities associated with PD-1/PD-L1 blockade cannot be ignored.

By unbalancing the immune system function, like other immune checkpoints, PD-1 inhibitors may lead to excessive inflammatory reactions termed as immune-related adverse events (irAEs), which cause collateral damage to any organ system such as the skin, pulmonary, gastrointestinal and so on^12^. Among irAEs noted during trials of PD-1 inhibitors, pneumonitis has been considered to be an “event of special interest”, occurring at a rate of around 3% and resulting in three treatment-related deaths in a phase I trial of nivolumab for NSCLC^13^. However, the morbidity and severity of pneumonitis in the treatment of other malignancies with PD-1 inhibitors are still obscure and need to be figured out. The appearance of pneumonitis is unpredictable and tends to occur later than the other irAEs, most commonly between 7.4 and 24.3 months after initiating therapy when some patients may have already discharged from hospital^14^,^15^. This late-onset autoimmune pneumonitis is considered to be more dangerous. Poor management of pneumonitis may lead to serious lung injury or even life-threatening consequences. Due to the lack of literature and sufficient data from large-sample clinical trials, it is difficult to thoroughly identify and understand the risk and extent of pneumonitis with PD-1 inhibitors. In this study, we reviewed selected published and presented randomized clinical trials investigating PD-1 inhibitors in multiple malignant tumor types, and conducted a meta-analysis on PD-1 inhibitor-related pneumonitis. The results would provide important information to clinicians and cause their attention on the pulmonary safety of anti-PD-1 immunotherapy.

## Materials and Methods

### Search strategy

We searched MEDLINE, EMBASE, the Cochrane Library and ClinicalTrials.gov (http://clinicaltrials.gov/) for the reported clinical trials. The latest date of the search was July 31, 2016. The search terms included: “PD-1”, “programmed cell death 1”, “immune checkpoint inhibitor”, “pembrolizumab”, “lambrolizumab”, “keytruda”, “MK-3475”, “SCH900475”, “nivolumab”, “opdivo”, “BMS-936558”, “MDX-1106”, “ONO-4538”, “randomized conctrolled trials”, or “clinical trials”. For details of the search strategy, please see the supplementary materials.

### Study selection

The selection of studies was conducted according to the Preferred Reporting Items for Systematic Reviews and Meta-Analyses (PRISMA) statement^16^. Clinical trials that met the following criteria were included:

- Randomized phase II, III, and IV trials
- Participants who received PD-1 inhibitors.
- Events or event rates and sample sizes available for pneumonitis.

Data on the pneumonitis were extracted from the safety profile of each clinical trial. The clinical endpoints were classified according to the Common Terminology Criteria for Adverse Events (CTCAE) of the National Cancer Institute^17^. Two investigators independently reviewed the title and abstract of each study. Articles that could not be decided based on title and abstract were retrieved for full-text review.

### Data extraction and quality assessment

The literature screening, data extraction and quality assessment of the trials were independently conducted by two reviewers (Jiaying Wu and Dongsheng Hong). If reviewers disagreed, a third reviewer (Jing Miao) would intervene until a consensus was reached. Structured data extraction forms were used to gather relevant data from each trial. The following information was extracted from every article: first author’s name, year of publication, journal, study type, underlying disease, trial phase, number of enrolled patients, treatment and control cohort, the number of patients with all-grade (grade 1–5) and high-grade (grade 3–5) pneumonitis (Table 1). The quality of the methodology in each trials was assessed by the Jadad criteria^18^. The quality of each included trials was graded, with high-quality trials classified as those with a score of ≥3 and low-quality trials classified as those with a score of ≤2.

### Data synthesis and analysis

All the meta-analyses were processed with R software, version 3.3.1 (The R Foundation for Statistical Computing, http://www.r-project.org). Data on patients with pneumonitis and on patients treated with PD-1 inhibitors were extracted from the safety profiles of all the included trials, and the proportions and 95% confidence intervals (CIs) of were calculated to assess the incidence. We used the Peto method to calculate OR and 95%CI because this method provided the best confidence interval coverage and was more powerful and relatively less biased when dealing with low event rates^19^. For trials with a control group, we calculated odds ratios (ORs) and 95% CIs. It was considered to be statistically significant when *P* < 0.05. The heterogeneity test was assessed by the *Q* statistic and *I*^2^ statistic. *I*^2^ > 40% indicated statistically significant heterogeneity and the statistical method should be changed to random-effects model. The analysis of subgroups was carried out according clinical characteristics. A statistical test with a *p*-value less than 0.05 was considered significant.

## Results

### Search results and population characteristics

**Figure 1:**
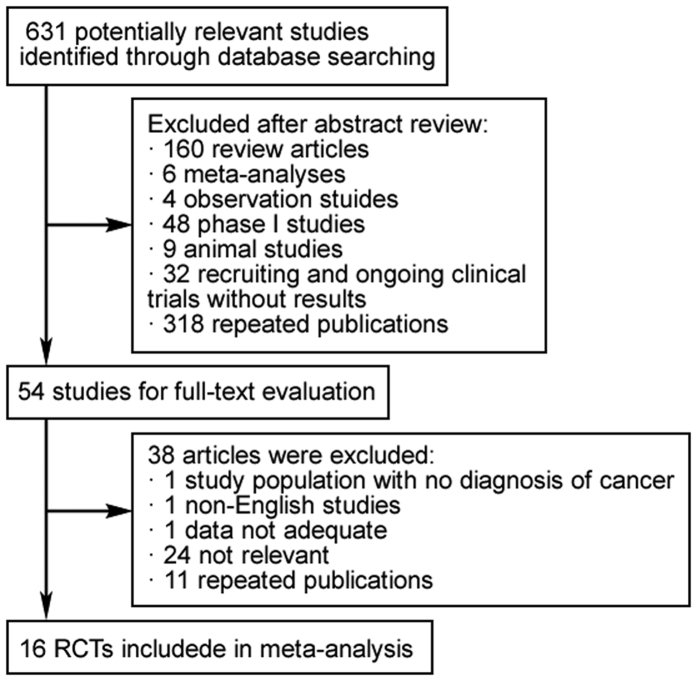
16 RCTs included in meta-analysis.

Our search yielded a total of 631 potentially relevant clinical trials with PD-1 inhibitors. After reviewing and screening, 16 primary studies which met our inclusion criteria^9^,^10^,^11^,^20^,^21^,^22^,^23^,^24^,^25^,^26^,^27^,^28^,^29^,^30^,^31^,^32^, including 6360 subjects, were pooled for the meta-analysis (Fig. 1). The baseline characteristics of each trial were shown in Table 2, containing 8 phase II clinical trials and 8 phase III clinical trials. Nine of them were randomized controlled trials (RCTs). Median sample size was 315 subjects (interquartile range, 122–741 subjects). Of note, two phase II trials of nivolumab in patients with ovarian cancer and Hodgkin’s lymphoma were excluded from this analysis owing to no available data on pneumonitis^33^,^34^. Of the sixteen trials included, five were blinded, and the rest were open-labelled. All the included RCTs were of high-quality with a Jadad score ranged from 3 to 5, while the score of the single-arm trials were only 1. According to the eligibility criteria of the majority of the trails, patients with impaired renal, hepatic or bone marrow function were excluded and most of the patients have Eastern Cooperative Oncology Group (ECOG) performance-status score of 0 or 1 (on a scale from 0 to 5, with higher scores indicating greater disability). The guidelines of the PRISMA statement were followed in this meta-analysis.

### Overall incidences of all-grade and high-grade pneumonitis

**Figure 2:**
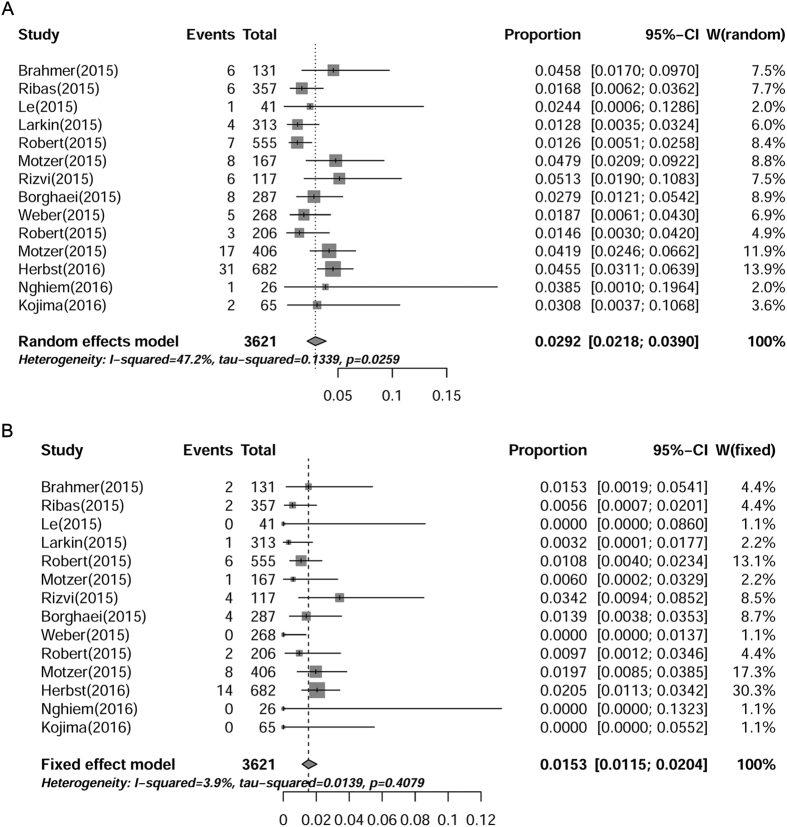
Annotated forest plot for meta-analysis of the incidence of pneumonitis in cancer patients who were assigned PD-1 inhibitors. Summary incidences of all-grade (**A**) and high-grade (**B**) pneumonitis were calculated with random effects model and fixed effects model, respectively. Size of squares was directly proportional to amount of information available.

A total of 6360 subjects treated with anti-PD-1 from 16 trials were included for analysis of all-grade and high-grade pneumonitis. Pneumonitis was one of the greatest concerns for anti-PD-1-related pulmonary adverse effects^14^,^35^,^36^. All-grade pneumonitis induced by PD-1 inhibitor monotherapy was observed in 14 of the 16 included studies and high-grade (grade 3–5) pneumonitis was only observed in 10 studies. The lowest incidence of pneumonitis was reported to be 0.72% in a phase III clinical trial of patient with advanced melanoma^23^, and the highest incidence was reported to be 4.7% in patient with advanced NSCLC^30^. As shown in Fig. 2A,B, the overall incidence of all- and high-grade pneumonitis was 2.92% (95% CI: 2.18–3.90%) and 1.53% (95% CI: 1.15–2.04%), respectively. Furthermore, four squamous NSCLC patients were reported to discontinue nivolumab treatment due to pneumonitis^20^ and one patient died from pneumonia^25^. These results demonstrated that pneumonitis induced by PD-1 inhibitors were not rare and worth noting.

### Incidences of all-grade and high-grade pneumonitis with the combination of nivolumab and ipilimumab

The incidence of pneumonitis associated with the combination of nivolumab and ipilimumab was 6.88% (95% CI: 5.00–9.39%) for all-grade, and 1.88% (95% CI: 0.98–3.57%) for high-grade, according to the fixed-effects model (Table 3). The morbidity of pneumonitis with combination therapy, especially all-grade pneumonitis, was significantly higher than that with monotherapy (all-grade: RR = 23.81, [95% CI: 15.38–35.71], *P* < 0.001; high-grade: RR = 1.23, [95% CI: 0.63–2.38], *P* = 0.58). This result demonstrated that the risk of pneumonitis from the combination therapy seemed to be more severe than that of anti-PD-1 monotherapy.

### ORs of pneumonitis for patients receiving PD-1 inhibitors compared with routine chemotherapy

**Figure 3:**
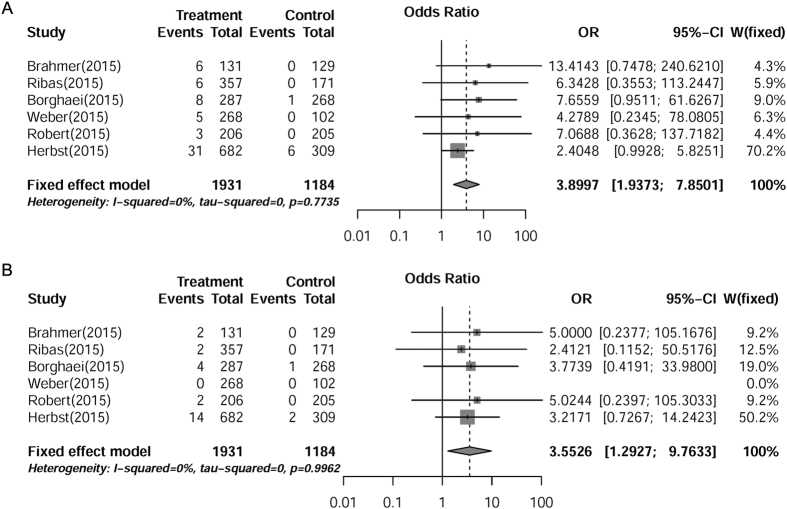
OR of PD-1 inhibitors-associated pneumonitis *versus* routine chemotherapy from six RCTs of patients with cancer. Summary ORs of all-grade (**A**) and high-grade (**B**) were calculated with fixed effects model. Size of squares was directly proportional to amount of information available.

We analyzed the OR of PD-1 inhibitors-induced pneumonitis compared with routine chemotherapy. The pooled OR demonstrated that PD-1 inhibitors significantly increased the risks of developing all-grade (OR = 3.90, [95% CI: 1.94–7.85], *P* = 0.0001) and high-grade (OR = 3.55, [95% CI: 1.29–9.76], *P* = 0.014) pneumonitis in cancer patients, according to the fixed effects model (Fig. 3A,B). These results indicated that the risk of pneumonitis induced by PD-1 inhibitors was much higher than that of routine chemotherapeutic agents in cancer patients.

### Subgroup analysis of incidences of pneumonitis in patients with different underlying malignancies

**Figure 4:**
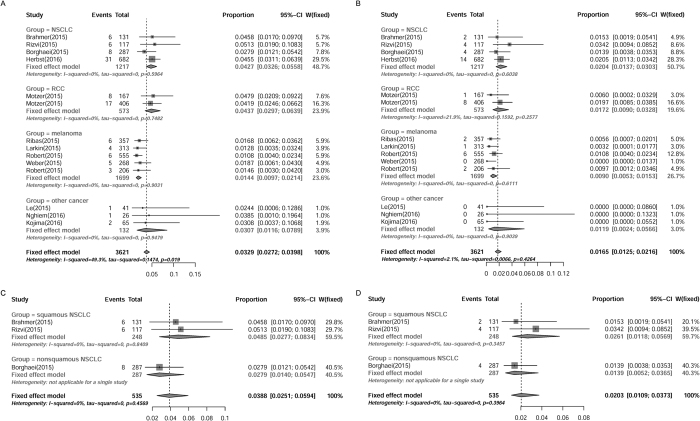
Subgroup analysis of incidences of PD-1 inhibitors-associated pneumonitis in patients with different tumor types. Summary incidences of all-grade (**A**) and high-grade (**B**) pneumonitis in patients with all the reported tumor types were calculated with fixed effects model. Incidences of all-grade (**C**) and high-grade (**D**) pneumonitis in patients with squamous NSCLC and non-squamous NSCLC were also calculated with fixed effects model. Size of squares was directly proportional to amount of information available.

Patients with NSCLC may be more susceptible to develop pneumonitis owing to the previous pulmonary dysfunction. The clinical trials included were stratified for subgroup analysis according to the type of tumor treated (NSCLC, melanoma, RCC and other malignancies). For patients with NSCLC, the overall incidence of all- and high-grade pneumonitis was 4.27% (95% CI: 3.26–5.58%) and 2.04% (95% CI: 1.37–3.03%), respectively, as determined by fixed effects model. Surprisingly, the incidence of pneumonitis in patient with RCC was as high as that of NSCLC (all-grade 4.37% [95% CI: 2.97–6.39%]; high-grade 1.72% [95% CI: 0.09–3.28%]). For patients with melanoma, the overall incidence of all- and high-grade pneumonitis was 1.44% (95% CI: 0.97–2.14%) and 0.90% (95% CI: 0.53–1.53%), respectively, which was the lowest among all the reported tumor types. The other reported cancers included Merkel-cell carcinoma, esophageal cancer, colorectal cancer and so on. For other cancer patients, the incidence of pneumonitis was a little bit lower than that of NSCLC or RCC (all-grade 3.07% [95% CI: 1.16–7.89%]; high-grade 1.19% [95% CI: 0.24–5.66%]) (Fig. 4A,B). As seen in Fig. 4C,D, the incidence of pneumonitis in patients with squamous NSCLC was higher than that with non-squamous NSCLC (all-grade: 4.85% *vs*. 2.79%; high-grade: 2.61% *vs*. 1.39%), though no significant difference was detected here (*P* > 0.0.5), probably because of the too small sample size. Hence, these results indicated that the morbidity of pneumonitis in patients with melanoma was the lowest among all the reported tumor types, while the NSCLC and RCC patients might be more susceptible to PD-1 inhibitors-induced pneumonitis.

### Subgroup analysis of incidences of pneumonitis in patients with different dosages of PD-1 inhibitors

**Figure 5:**
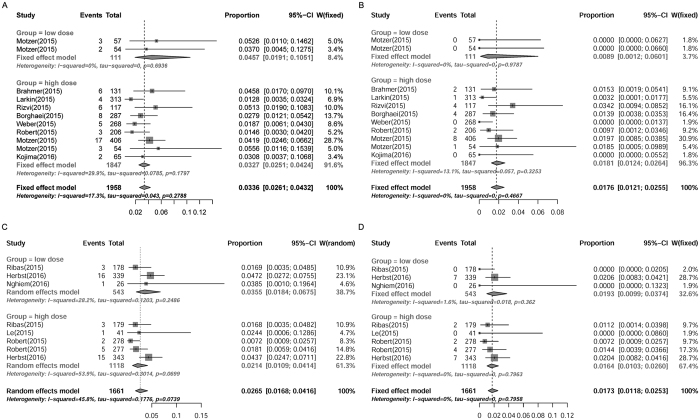
Subgroup analysis of incidences of pneumonitis in cancer patients receiving different doses of PD-1 inhibitors. Summary incidences of all-grade (**A**) and high-grade (**B**) pneumonitis in patients receiving different doses of nivolumab were calculated with fixed effects model. Incidences of all-grade (**C**) and high-grade (**D**) pneumonitis in patients receiving different doses of pembrolizumab were calculated with random effects model and fixed effects model, respectively. Size of squares was directly proportional to amount of information available.

To explore whether the risk of anti-PD-1-induced pneumonitis was dose-dependent, another subgroup analysis was conducted. Considering the different doses and frequencies of administration of PD-1 inhibitors in different clinical trials, the dosage subgroups were distributed and defined as follows: low-dose group (<5 mg/kg, every six weeks), high-dose group (>5 mg/kg, every six weeks). The incidences of all-grade pneumonitis for different dosage groups were 4.57% (95% CI: 1.91–10.51%) for low-dose of nivolumab, 3.27% (95% CI: 2.51–4.24%) for high-dose of nivolumab (Fig. 5A), and 3.55% (95% CI: 1.84–6.75%) for low-dose of pembrolizumab, 2.14% (95% CI: 1.09–4.14%) for high-dose of pembrolizumab (Fig. 5C). The incidences of high-grade pneumonitis for different dosage groups were 0.89% (95% CI: 0.12–6.01%) for low-dose of nivolumab, 1.81% (95% CI: 1.24–2.64%) for high-dose of nivolumab (Fig. 5B), and 1.93% (95% CI: 0.99–3.74%) for low-dose of pembrolizumab, 1.64% (95% CI: 1.03–2.60%) for high-dose of pembrolizumab (Fig. 5D). No significant differences were detected between the low-dose and high-dose groups of either nivolumab or pembrolizumab for all-grade (nivolumab: RR = 1.40, [95% CI: 0.57–3.41], *P* = 0.47; pembrolizumab: RR = 1.66, [95% CI: 0.65–4.21], *P* = 0.10) and high-grade (nivolumab: RR = 0.49, [95% CI: 0.067–3.61], *P* = 0.49; pembrolizumab: RR = 1.18, [95% CI: 0.52–2.65], *P* = 0.54). These results indicated that the risk of pneumonitis evoked by PD-1 inhibitors was likely to be independent of the administration dosages.

### Subgroup analysis of incidences of pneumonitis in patients receiving nivolumab or pembrolizumab

**Figure 6:**
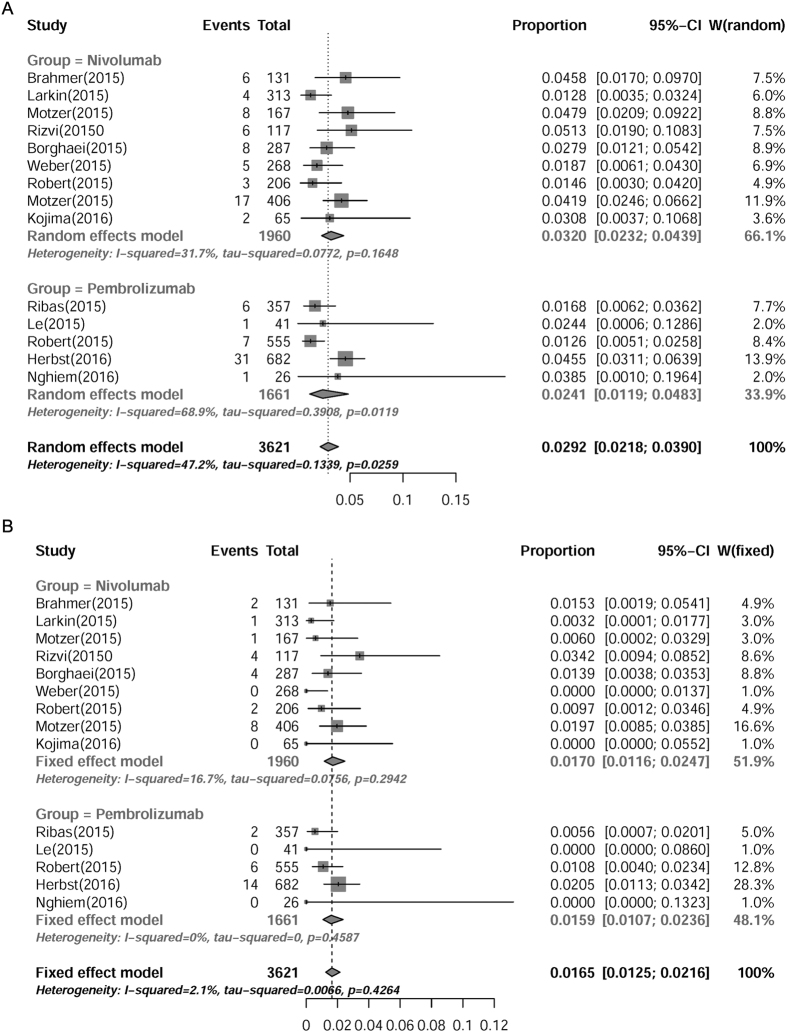
Subgroup analysis of incidences of pneumonitis in cancer patients receiving nivolumab or pembrolizumab. Summary incidences of all-grade (**A**) and high-grade (**B**) pneumonitis in patients receiving either nivolumab or pembrolizumab were calculated with random effects model and fixed effects model, respectively. Size of squares was directly proportional to amount of information available.

Although both nivolumab and pembrolizumab target PD-1 receptor, the chemical structures of these two mAbs are different from each other. Therefore, we further compared the potential risks of treatment-related pneumonitis between nivolumab and pembrolizumab. The incidence of nivolumab-associated pneumonitis was 3.20% (95% CI: 2.32–4.39%) for all-grade, and 1.70% (95% CI: 1.16–2.47%) for high-grade, while the incidence of pembrolizumab-associated pneumonitis was 2.41% (95% CI: 1.19–4.83%) for all-grade, and 1.59% (95% CI: 1.07–2.36%) for high-grade (Fig. 6A,B). The incidence of treatment-related pneumonitis, either all-grade (RR = 1.33, [95% CI: 0.62–2.88], *P* = 0.15) or high-grade (RR = 1.07, [95% CI: 0.62–1.85], *P* = 0.78), showed no significant differences between patients receiving nivolumab and pembrolizumab. These results suggested that the risks of PD-1 inhibitors-associated pneumonitis were similar between nivolumab and pembrolizumab.

## Discussion

In general, the PD-1 checkpoint inhibitors are well-tolerated and only with mild and common adverse reactions including fatigue, rash, diarrhea, nausea and pruritus^37^. Nevertheless, PD-1 inhibitors were also documented with severe pneumonitis, which could lead to cessation of treatment^38^. Until now, the incidence of pneumonitis with PD-1 inhibitors treatment has not been fully evaluated. Here we reported the results from a meta-analysis on PD-1 inhibitors-associated autoimmune pneumonitis, based on 16 clinical trial including 6360 cancer patients. Our results indicated that: 1. Anti-PD-1 immunotherapy would significantly increase the incidence and severity of pneumonitis compared with routine chemotherapy in cancer patients; 2. The frequencies and severities of PD-1 inhibitors-mediated pneumonitis were quite different in patients with various tumor types; 3. The appearance of PD-1 inhibitors-related pneumonitis was not associated with its dosage. 4. The potential risks of treatment-associated pneumonitis were similar between nivolumab and pembrolizumab. Pidilizumab, another mAb being developed by Medivation, originally designed to bind to PD-1, was currently uncertain^39^, so the data on pidilizumab were excluded from our analysis.

Our analysis showed that the incidence of all-grade pneumonitis in patients receiving PD-1 inhibitors was 2.92% (95% CI: 2.18–3.90%), similar with previous studies^12^,^14^,^35^. However, the incidence of high-grade pneumonitis secondary to anti-PD-1 treatment was previously considered to be <1%^40^, whereas our data showed it should be higher to 1.53% (95% CI: 1.15–2.04%). This difference might be due to the small sample bias from a spot of trials, and the bias could be eliminated through meta-analysis as far as possible. This result indicated that the risk of high-grade pneumonitis might have been underestimated for a long time and deserved more attention during the future PD-1 immunotherapy. Compared with routine chemotherapy such as docetaxel, dacarbazine, paclitaxel and carboplatin, patients treated with PD-1 inhibitors were more susceptible to pneumonitis (all-grade: OR = 3.90, [95% CI: 1.94–7.85], *P* = 0.0001; high-grade: OR = 3.55, [95% CI: 1.29–9.76], *P* = 0.014), suggesting that the risk of pneumonitis induced by anti-PD-1 immunotherapy is higher than that of most chemotherapy. Moreover, the combination of anti-CTLA-4 and anti-PD-1 was likely to be associated with higher incidence of pneumonitis than anti-PD-1 monotherapy (6.88% *vs*. 2.92%, *P* < 0.001). In consideration of the cumulative toxicities, combination of two or more immunotherapies should be implemented with special caution.

By comparing the different tumor types involved, one review deemed that the rate of grade 3–4 pneumonitis induced by PD-1 inhibition was similar^15^. In contrast, our statistical analysis suggested that the overall incidence of pneumonitis related with PD-1 inhibitors was actually much lower in melanoma patients than many other tumor types, implicating a wide safety range of PD-1 inhibitors in melanoma treatment. The highest incidence of pneumonitis was observed in NSCLC and RCC patients. As lungs were core organs that often already impaired in NSCLC patients, it was generally accepted that the immune-mediated pneumonitis was more likely to attack patients with NSCLC. Surprisingly, the morbidity of all- or high-grade pneumonitis in RCC patients was as high as that of NSCLC. It was recently reported that the prior high-dose chemotherapy might be associated with the development of PD-1 inhibitors-related pneumonitis, because the patients with prior heavy chemotherapy seemed to have a much earlier onset of pneumonitis at approximately one month of therapy (regular onset time was over three weeks)^41^. Since the inclusion criteria of the RCTs on RCC included at least one prior anti-tumor therapy, while those of the other RCTs were no more than one systemic treatment, this difference might partly result in the high incidence of PD-1 inhibitors-related pneumonitis in RCC patients. Nevertheless, the two RCTs on RCC both were for nivolumab. More new information of pembrolizumab in RCC patients would be welcome to further characterize the spectrum of clinical manifestations of PD-1 immunotherapy.

In addition, higher incidence of pneumonitis was also found in patients with squamous NSCLC compared with those with non-squamous NSCLC (all-grade: 4.85% *vs*. 2.79%; high-grade: 2.61% *vs*. 1.39%), but this conclusion needed to be further tested by more large-sample RCTs. In our analysis, there was only one death due to pneumonitis secondary to anti-PD-1 treatment, who was a patient with advance, refractory squamous NSCLC^25^. Furthermore, as reported in a phase II/III clinical trial^30^, pembrolizumab showed more significant benefits to non-squamous NSCLC patients compared with squamous NSCLC patients, implicating that non-squamous NSCLC might be a more suitable indication for PD-1 inhibitors with higher efficiency and lower toxicity.

Generally speaking, the irAEs were supposed to be cumulative and dose-dependent as seen in anti-CTLA-4 treatment^42^,^43^, so it was always a tough task of choosing the optimal dosage to balance between efficiency and safety. Surprisingly, in the subgroup analysis, we found that the morbidity and severity of pneumonitis was independent of the administration dosages with either nivolumab or pembrolizumab. The incidences of all-/high-grade pneumonitis in the two dosage subgroups were 4.57%/0.89% for low-dose groups of nivolumab, 3.27%/1.81% for high-dose group of nivolumab (both *P* > 0.05), and 3.55%/1.93% for low-dose group of pembrolizumab, 2.14%/1.64% for high-dose group of pembrolizumab (both *P* > 0.05), respectively, implying a much broader range of feasible and secure dosages in the anti-PD-1 treatment. In an initial single-dose phase I study, PD-1 occupancy (by nivolumab) appeared to be dose-independent, with a mean plateau occupancy of 72% (59% ~ 81%) observed over 57 days following infusion^44^. This pharmacodynamic feature of PD-1 inhibitor may help to explain why the incidence of treatment-related pneumonitis was dose-independent. Moreover, the dose regimens for the two PD-1 inhibitors are different. Pembrolizumab is administered via continuous *i.v.* infusion over 30 minutes at an optimal dose of 10 mg/kg of actual body weight every three weeks, while nivolumab is administered as a continuous *i.v.* infusion over 60 minutes at a recommended dose of 3 mg/kg of actual body weight every two weeks. Although *P* > 0.5, the incidence of pneumonitis induced by nivolumab for either all- or high-grade was a little bit higher than that of pembrolizumab (3.20% *vs.* 2.41%, 1.70% *vs.* 1.59%). In view of the higher incidence of pneumonitis induced by sequential administration of nivolumab and ipilimumab (Table 3), it is possible that the frequency of exposure of checkpoint inhibitors would play an important role on the onset of treatment-related pneumonitis, rather than the dose intensity.

Recently, a polymorphism in a regulatory site of the PD-1 gene was reported to be associated with increased risk of autoimmune diseases in humans such as rheumatoid arthritis (RA). Some single-nucleotide polymorphisms (SNP) such as PD-1.1 G/A and PD-1.3 GA were found to be involved in the RA susceptibility^45^,^46^. Moreover, previous studies also indicated that immune-mediated pneumonitis may be associated with autoimmune process^47^,^48^. Animal experimental results demonstrated that pneumonitis was concomitant with RA and even shared the same immunity mechanism with RA^49^,^50^. The same CD8(+) T cell clones from arthritic lesions of mice could elicit both synovitis and pneumonitis, suggesting the tight correlation between pneumonitis and RA. Considering the association of PD-1 polymorphisms with the development of RA, it is quite possible that genetic polymorphisms of PD-1 would also be relevant to the susceptibility of PD-1 inhibitors-related pneumonitis.

Medication-induced pneumonitis is often deemed reversible, but occasionally could be fatal, and the timing of onset varies as well. The early diagnosis and prompt treatment for immune-mediated pneumonitis is usually difficult, because it is challenging to definitely differentiate among infection, early pulmonary edema, immune-related pneumonitis, immune-mediated tumor inflammation, and even tumor progression, especially in patients with lung cancer^51^. Alerting symptoms include dyspean, cough, fever, chest pain, and fine inspiratory crackles^14^,^52^. The treatment of pneumonitis is mainly based on the steroids^52^. Mild cases (grade 1) could be managed by withholding therapy. Higher grade cases may be managed with oral or intravenous corticosteroids, while severe cases (grade 3–4) should be discontinued immunotherapy and hospitalized for intravenous corticosteroids. If a course of corticosteroids is unable to control the initial symptoms, additional immunosuppressants such as infliximab, cyclophosphamide, and mycophenolate mofetil, could be considered as well^12^,^36^,^51^.

Our meta-analysis has a few limitations. First, because of the short time to market, the number of clinical trials for PD-1 inhibitors is not large enough to fully evaluate the efficiency and safety of PD-1 inhibitors, especially lack of RCTs with high-quality. So far, we have retrieved dozens of ongoing or recruiting clinical trials which would further extend our knowledge of the spectrum of manifestations and management of autoimmune pneumonitis associated with PD-1 inhibitors. Second, different doses and frequencies of administration of PD-1 inhibitors were used in different clinical trials, and the baseline characteristics of patients also varied between studies. We have tried to overcome the heterogeneity through subgroup analysis. Third, although the statistical data in this study demonstrated that the incidence of pneumonitis was different among tumor types, the cause(s) of differences was still unclear. Possible confound factors such as smoking and genetic polymorphism might be involved. More clinical studies are needed to further address the possible confound factors. Fourth, our analysis was conducted at the study level rather than individual patient data (IPD) level, meaning the potential variables at the patient level were not involved in the analysis.

## Conclusion

The data of our meta-analysis demonstrated that PD-1 inhibitors dramatically increased the risk of immune-associated pneumonitis in cancer patients compared with routine chemotherapy. The onset of pneumonitis was independent of the dosage of either nivolumab or pembrolizumab. Relatively, under PD-1 immunotherapy, melanoma patients might have the minimum risk of treatment-associated pneumonitis, while NSCLC and RCC patients should be rather cautious for the appearance of pneumonitis. These results would provide considerable information for clinicians in managing this rare but potentially life-threaten toxic effect.

## Additional Information

**How to cite this article:** Wu, J. *et al*. PD-1 inhibitors increase the incidence and risk of pneumonitis in cancer patients in a dose-independent manner: a meta-analysis. *Sci. Rep.* **7**, 44173; doi: 10.1038/srep44173 (2017).

**Publisher's note:** Springer Nature remains neutral with regard to jurisdictional claims in published maps and institutional affiliations.

## Supplementary Material

Supplementary Information

## Figures and Tables

**Table 1:** Grading of pneumonitis as per CTCAE.

Grade	Clinical description
1	Asymptomatic; clinical or diagnostic observations only; intervention not indicated
2	Symptomatic; medical intervention indicated; limiting instrumental activities of daily living (ADL)
3	Severe symptoms; limiting self-care ADL; oxygen indicated
4	Life-threatening respiratory compromise; urgent intervention indicated (e.g., tracheotomy or intubation)
5	Death

**Table 2:** Main characteristics and results of the eligible studies.

Study	Year	Phase	Disease	Treatment	No. of patients with pneumonitis		No. of Patients enrolled	Jadad Score
					All- grade	High- grade		
Brahmer	2015	III	NSCLC	nivolumab 3 mg/kg, Q2W	6	2	131	3
				docetaxel 75 mg/m^2^, Q3W	0	0	129	
Ribas	2015	II	melanoma	pembrolizumab 2 mg/kg, Q3W	3	0	178	4
				pembrolizumab 10 mg/kg, Q3W	3	2	179	
				investigator-choice chemotherapy	0	0	171	
Le	2015	II	progressive metastatic carcinoma	pembrolizumab 10 mg/kg, Q2W	1	0	41	1
Larkin	2015	III	melanoma	nivolumab 3 mg/kg, Q2W	4	1	313	4
				ipilimumab 3 mg/kg, Q3W followed by nivolumab 3 mg/kg, Q2W	20	3	313	
				ipilimumab 3 mg/kg, Q3W	5	1	311	
Postow	2015	II	melanoma	ipilimumab 3 mg/kg, Q3W followed Nivolumab 3 mg/kg, Q2W	10	3	94	5
				ipilimumab 3 mg/kg, Q3W	2	1	46	
Robert	2015	III	melanoma	pembrolizumab 10 mg/kg, Q2W	2	2	278	3
				pembrolizumab 10 mg/kg, Q3W	5	4	277	
				ipilimumab 3 mg/kg, Q3W	2	2	256	
Motzer	2015	II	RCC	nivolumab 0.3 mg/kg, Q3W	3	0	59	3
				nivolumab 2 mg/kg, Q3W	2	0	54	
				nivolumab 10 mg/kg, Q3W	3	1	54	
Rizvi	2015	II	NSCLC	nivolumab 3 mg/kg, Q2W	6	4	117	1
Borghaei	2015	III	NSCLC	nivolumab 3 mg/kg, Q2W	8	4	287	3
				docetaxel 75 mg/m^2^, Q3W	1	1	268	
Weber	2015	III	melanoma	nivolumab 3 mg/kg, Q2W	5	0	268	5
				investigator-choice chemotherapy	0	0	102	
Robert	2015	III	melanoma	nivolumab 3 mg/kg, Q2W	3	2	206	3
				dacarbazine 1000 mg/m^2^, Q3W	0	0	205	
Motzer	2015	III	RCC	nivolumab 3 mg/kg, Q2W	17	8	406	3
				everolimus 10 mg/kg, orally QD	55	12	397	
Herbst	2016	II/III	NSCLC	pembrolizumab 2 mg/kg, Q3W	16	7	339	3
				pembrolizumab 10 mg/kg, Q3W	15	7	343	
				docetaxel 75 mg/m^2^, Q3W	6	2	309	
Nghiem	2016	II	Merkel-cell carcinoma	pembrolizumab 2 mg/kg, Q3W	1	0	26	1
Weber	2016	II	melanoma	nivolumab 3 mg/kg, Q2W followed ipilimumab 3 mg/kg, Q3W	4	3	68	3
				ipilimumab 3 mg/kg, Q3W followed nivolumab 3 mg/kg, Q2W	2	0	70	
Kojima	2016	II	esophageal cancer	nivolumab 3 mg/kg, Q2W	2	0	65	1

**Table 3:** Summary incidences of pneumonitis in cancer patients receiving combination therapy with PD-1 inhibitor and CTLA-4 inhibitor.

Outcome	No. of Studies	No. of Participants	Incidence (95% CI)	Heterogeneity: *P*: I^2^	Model
All-grade pneumonitis	3	36/545	6.88% (5.00–9.39%)	0.1733; 42.9%	Fixed effects model
High-grade pneumonitis	3	9/545	1.88% (0.98–3.57%)	0.3157; 13.3%	Fixed effects model
