# Supplementary material for: PD-1 inhibitors increase the incidence and risk of pneumonitis in cancer patients in a dose-independent manner: a meta-analysis
Source: Sci Rep. 2017 Mar 8;7:44173. doi: 10.1038/srep44173 (PMC5341153; doi:10.1038/srep44173)
Supplement: Supplementary Information [file srep44173-s1.doc]

**PD-1 inhibitors increase the incidence and risk of pneumonitis in cancer patients in a dose-independent manner: a meta-analysis**

Jiaying Wu^1+^, Dongsheng Hong^1+^, Xiangnan Zhang^2^, Xiaoyang Lu^1^ and Jing Miao^1*^

^1^The First Affiliated Hospital, College of Medicine, Zhejiang University, Hangzhou, China

^2^ Department of Pharmacology, College of Pharmaceutical Sciences, Zhejiang University, Hangzhou, China

These two authors contributed equally to this work.

***Corresponding author:** Dr. Jing Miao, Ph.D. The First Affiliated Hospital, College of Medicine, Zhejiang University, Hangzhou, China, #79 Qingchun Road, Hangzhou 310003, China.

**Tel:** +86 571 8723 6675, **Fax:** +86 571 8723 6675, **E-mail:** [joemj1005@163.com](mailto:joemj1005@163.com)

PubMed

| Search | Query | Items found |
| --- | --- | --- |
| #1 | Search pembrolizumab[Title/Abstract] | 344 |
| #2 | Search lambrolizumab[Title/Abstract] | 20 |
| #3 | Search Keytruda[Title/Abstract] | 15 |
| #4 | Search MK-3475[Title/Abstract] | 30 |
| #5 | Search nivolumab[Title/Abstract] | 524 |
| #6 | Search Opdivo[Title/Abstract] | 19 |
| #7 | Search MDX-1106[Title/Abstract] | 3 |
| #8 | Search BMS-936558[Title/Abstract] | 21 |
| #9 | Search ONO-4538[Title/Abstract] | 6 |
| #10 | Search anti-PD-1[Title/Abstract] | 548 |
| #11 | Search anti-programmed cell death 1 receptor [Title/Abstract] | 2 |
| #12 | Search (#1 OR #2 OR #3 OR #4 OR #5 OR #6 OR #7 OR #8 OR #9 OR #10 OR #11) | 1103 |
| #13 | Search randomized controlled trial[Publication Type] | 416177 |
| #14 | Search controlled clinical trial[Publication Type] | 501927 |
| #15 | Search randomized[Title/Abstract] | 383183 |
| #16 | Search placebo[Title/Abstract] | 177270 |
| #17 | Search clinical trials as topic[mesh: noexp] | 176230 |
| #18 | Search randomly[Title/Abstract] | 256347 |
| #19 | Search trial[Title] | 154592 |
| #20 | Search (#13 OR #14 OR #15 OR #16 OR #17 OR #18 OR #19 ) | 1041999 |
| #21 | Search (animals [mh] NOT humans [mh]) | 4236012 |
| #22 | Search (#20 NOT #21) | 960611 |
| #23 | #12 AND #22 | 120 |

1. EMBASE

| Search | Query | Items found |
| --- | --- | --- |
| #1 | ‘pembrolizumab’:ab,ti | 548 |
| #2 | ‘lambrolizumab’:ab,ti | 22 |
| #3 | ‘keytruda’:ab,ti | 17 |
| #4 | ‘mk 3475’:ab,ti | 169 |
| #5 | ‘nivolumab’:ab,ti | 792 |
| #6 | ‘opdivo’:ab,ti | 17 |
| #7 | 'mdx 1106':ab,ti | 30 |
| #8 | 'bms 936558':ab,ti | 128 |
| #9 | 'ono 4538':ab,ti | 90 |
| #10 | 'ani pd 1':ab,ti | 1137 |
| #11 | ‘anti programmed cell death 1 receptor’:ab,ti | 1 |
| #12 | #1 OR #2 OR #3 OR #4 OR #5 OR #6 OR #7 OR #8 OR #9 OR #10 OR #11 | 1993 |
| #13 | 'crossover procedure'/exp | 47443 |
| #14 | 'double blind procedure'/exp | 130056 |
| #15 | 'randomized controlled trial'/exp | 407531 |
| #16 | 'single blind procedure'/exp | 22410 |
| #17 | random* | 1264163 |
| #18 | crossover* | 77896 |
| #19 | placebo* | 377222 |
| #20 | assign* | 290384 |
| #21 | allocat* | 118400 |
| #22 | volunteer* | 217763 |
| #23 | doubl* NEAR/5 blind* | 208983 |
| #24 | singl* NEAR/5 blind* | 37073 |
| #25 | factorial* | 56554 |
| #26 | #13 OR #14 OR #15 OR #16 OR #17 OR #18 OR #19 OR #20 OR #21 OR #22 OR #23 OR #24 OR #25 | 1938840 |
| #27 | #12 AND #26 | 425 |

1. **Cochrane Library**

| Search | Query |  |
| --- | --- | --- |
| #1 | pembrolizumab:ti,ab | 24 |
| #2 | lambrolizumab:ti,ab | 1 |
| #3 | keytruda:ti,ab | 1 |
| #4 | MK-3475:ti,ab | 16 |
| #5 | nivolumab:ti,ab | 42 |
| #6 | Opdivo:ti,ab | 1 |
| #7 | MDX-1106:ti,ab | 2 |
| #8 | BMS-936558:ti,ab | 6 |
| #9 | ONO-4538:ti,ab | 3 |
| #10 | anti-PD-1:ti,ab | 20 |
| #11 | anti-programmed cell death 1 receptor:ti,ab | 1 |
| #12 | {or #1-#11} | 75 |

1. **ClinivalTrials.gov**

| Search | Query |  |
| --- | --- | --- |
| #1 | "pembrolizumab" OR "lambrolizumab" OR "Keytruda" OR "MK-3475" OR "nivolumab" OR "Opdivo" OR "BMS-936558" OR "ONO-4538" OR "MDX-1106" OR "anti-PD-1" | 588 |
| #2 | Studies with results |  |
| #3 | 1# AND 2# | 11 |
